# Preparation of stable magnetic nanofluids containing Fe_3_O_4_@PPy nanoparticles by a novel one-pot route

**DOI:** 10.1186/1556-276X-6-230

**Published:** 2011-03-16

**Authors:** Baobao Zhao, Zhaodong Nan

**Affiliations:** 1 College of Chemistry and Chemical Engineering, Yangzhou University, Yangzhou, 225002, People's Republic of China

## Abstract

Stable magnetic nanofluids containing Fe_3_O_4_@Polypyrrole (PPy) nanoparticles (NPs) were prepared by using a facile and novel method, in which one-pot route was used. FeCl_3_·6H_2_O was applied as the iron source, and the oxidizing agent to produce PPy. Trisodium citrate (Na_3_cit) was used as the reducing reagent to form Fe_3_O_4 _NPs. The as-prepared nanofluid can keep long-term stability. The Fe_3_O_4_@PPy NPs can still keep dispersing well after the nanofluid has been standing for 1 month and no sedimentation is found. The polymerization reaction of the pyrrole monomers took place with Fe^3+ ^ions as the initiator, in which these Fe^3+ ^ions remained in the solution adsorbed on the surface of the Fe_3_O_4 _NPs. Thus, the core-shell NPs of Fe_3_O_4_@PPy were obtained. The particle size of the as-prepared Fe_3_O_4_@PPy can be easily controlled from 7 to 30 nm by the polymerization reaction of the pyrrole monomers. The steric stabilization and weight of the NPs affect the stability of the nanofluids. The as-prepared Fe_3_O_4_@PPy NPs exhibit superparamagnetic behavior.

## Introduction

Nanofluids, which contain nanoparticles dispersed in base fluids, have been proposed as a new kind of heat transfer medium because they can improve the heat transport and energy efficiency and may have potential applications in the field of heat transfer enhancement [1]. Magnetic nanofluids, suspension containing magnetic nanoparticles, such as magnetite (Fe_3_O_4_), iron (Fe), nickel (Ni), and cobalt (Co), show both magnetic and fluid properties and have important applications in industries [2,3]. Fe_3_O_4 _nanoparticles are always used to form magnetic nanofluids because of their broad potential and practical technological applications and fundamental scientific significance [3-10]. Recently, heat transfer enhancements were reported for γ-Fe_2_O_3 _magnetic nanofluids [3]. However, long-term stability of nanofluids is a major concern for the engineering applications [11-13]. Nanoparticles tend naturally to aggregate and sediment in the base fluid. Also, stable solution with large volume concentration is not easy to obtain. Therefore, technique breakthroughs are needed to produce well-dispersed and long-term stable nanofluids. Use of functionalized nanoparticles is a promising approach to achieve long-term stability of nanofluid [2].

Organic-inorganic nanocomposites with an ordered structure show new functional hybrids of organic and inorganic materials. Incorporation of nanosized particles in organic polymeric materials has been extensively studied because they combine the advantages of the inorganic materials and the organic polymers. Moreover, new properties of these hybrids can also show up because of synergetic effects, which can be scarcely obtained from the individual components. Combination of various conducting polymers with magnetic iron-oxides is more and more intensively investigated, because materials possessing both high magnetic susceptibility and high conductivity can be used in different applications, such as nonlinear optics, electrical, and magnetic shielding, magnetic electrocatalysis, and as microwave absorbers [14].

This study reports on the synthesis of stable nanofluids with a novel one-pot hydrothermal route, in which Fe_3_O_4 _coated by PPy NPs were contained. FeCl_3_·6H_2_O was used as the iron source and the oxidant to polymerize pyrrole monomers. Trisodium citrate was used as the reducing reagent. The procedure was carried out at low temperature and without any protection atmosphere. To our knowledge, this is the first report to direct synthesis of Fe_3_O_4_@PPy nanofluids. The mechanism of suspension was proposed.

## Experimental

Pyrrole monomer, iron (III) chloride hexahydrate (FeCl_3_·6H_2_O), sodium hydroxide (NaOH), trisodium citrate (Na_3_cit), and sodium dodecyl sulfate (SDS) were purchased from Sinopharm Chemical Reagent Company. Pyrrole monomer was distilled under reduced pressure, and other reagents were of analytical grade and used as received without further treatment. All solutions were prepared with twice-distilled water.

In a typical experiment, NaOH 0.0800 g (2.0 mmol) and 0.0230 g SDS (0.008 M) were dissolved into a 9 mL aqueous solution containing 0.1 M Na_3_cit (C_6_H_5_Na_3_O_7_·2H_2_O) under constant stirring. 1.0 mL of FeCl_3 _solution (1.0 M) and 0.3 mL pyrrole monomer were added into the solution. The whole mixture was stirred vigorously for 5 min to give a homogeneous solution. Subsequently, the solution was transferred into a 50 mL Teflon-lined stainless steel autoclave, and maintained at 160°C for 24 h. Afterward, the autoclave was allowed to cool down to room temperature naturally, and the resulting black Fe_3_O_4_@PPy nanofluid was produced. The deposit was obtained by magnetic separation method, and washed with distilled water and absolute ethanol for several times. The as-prepared black precipitate was dried under vacuum at 50°C for 24 h.

X-ray powder diffraction (XRD) measurements were performed on a Bruker D8 Advance X-ray Diffractometer with Cu Kα radiation (λ = 1.5418 Ǻ). The 2θ range used in the measurement was from 20° to 70°. Standard transmission electron microscopy (TEM) measurements were performed on a JEOL-2010 TEM at an acceleration voltage of 200 kV. Samples were first ultrasonically dispersed in deioned water and drop-cast onto copper grids. Magnetic characterization was carried out at room temperature using a vibrating sample magnetometer (VSM, Lakeshore 7307, USA). Infrared spectrum (FT-IR) measurements were performed on a Nicolet Aexus 470, with scanning from 4000 to 400 cm^-1 ^by using KBr pellets under ambient temperature.

## Results and discussion

### Stability of the as-synthesized Fe_3_O_4_@PPy nanofluids

**Figure 1:**
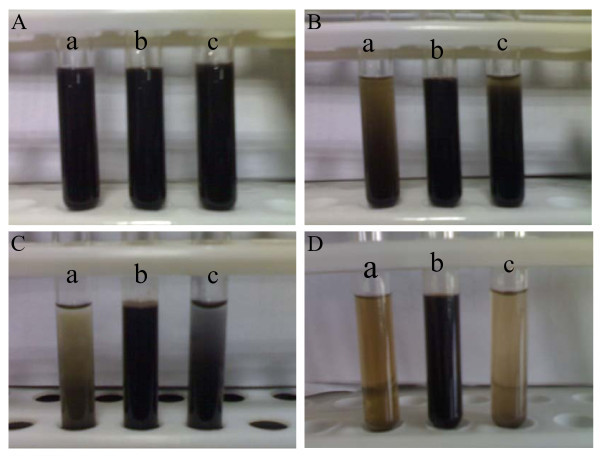
**Pictures of the nanofluids containing Fe_3_O_4_@PPy NPs standing for various times prepared under different amounts of pyrrole monomer at 160°C**: **(A) **Commencement, **(B) **1 h, **(C) **4 h, **(D) **1 week; (a) 0.1 mL, (b) 0.3 mL, (c) 0.5 mL.

Well-dispersed nanofluid can be prepared as shown in Figure 1A(b). The nanoparticles can still keep dispersing well after the nanofluid has been standing for 1 week and no sedimentation was observed as shown in Figure 1D(b), in which the nanoparticles were synthesized with 0.3 mL pyrrole monomer. This kind of nanofluid can be stable for more than 1 month. In order to investigate the effect of amount of pyrrole monomer on stability of the nanofluid, various amounts of pyrrole were added into the reaction system. Figure 1a,c shows the nanofluids with 0.1 and 0.5 mL pyrrole, respectively. Sedimentations can be clearly found after the nanofluids have been standing for 1 and 4 h as shown in Figure 1B(a, c) and 1C(a, c), respectively. When no pyrrole was added into the reaction solution, no stable was prepared (the figure is not shown here). These results proved that the most stable nanofluid was produced with 0.3 mL pyrrole.

### Effects of different amounts of pyrrole monomer on the structure and size of the as-prepared nanoparticles

**Figure 2:**
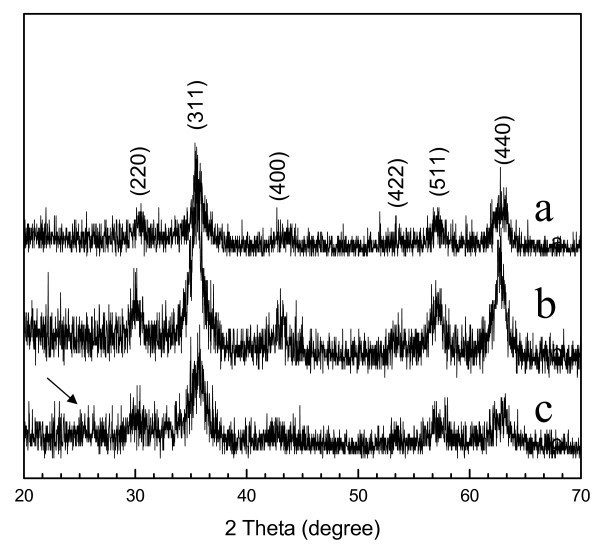
**XRD diffraction patterns of the Fe_3_O_4_@PPy deposits prepared under different amounts of pyrrole monomer at 160°C**: (a) 0.1 mL, (b) 0.3 mL, (c) 0.5 mL.

Figures 2 shows the XRD patterns of the products prepared with different amounts of pyrrole monomer. The XRD pattern of the sample produced without pyrrole is shown in Additional file 1, Figure S1. The broad peak appears at a 2θ value of 20°-30° in Figure 2c as the arrow's direction indexed PPy. No broad peak was clearly found in Figure 2a,b because of the small amount of pyrrole monomer added into the reaction system and the strong peaks of Fe_3_O_4_. Other major peaks at about 30°, 35°, 43°, 53°, 57°, and 62° were observed and could be assigned to diffraction from the (220), (311), (400), (422), (511), and (440) planes of Fe_3_O_4 _(JCPDS card no.79-0418), respectively [15]. Nevertheless, as magnetite (Fe_3_O_4_) and maghemite (γ-Fe_2_O_3_) havethe inverse spinel structure and have very similar XRD patterns, precise studies on the phase composition of the as-synthesized products were required. Using the formula 2d*sinθ = n*λ to calculate *d*, and compare to the standard numerical value [16], these as-prepared products are all magnetite (Fe_3_O_4_). These data are listed in Table 1.

**Table 1:** Comparison of *d*-spacing values of the as-synthesized samples with standard JCPDS Fe_3_O_4 _data

2Theta	Calculating data	Standard data
30.06	2.97	2.967
35.6	2.521	2.532
43.22	2.092	2.099
53.48	1.72	1.714
57.24	1.609	1.615
62.78	1.479	1.484

**Figure 3:**
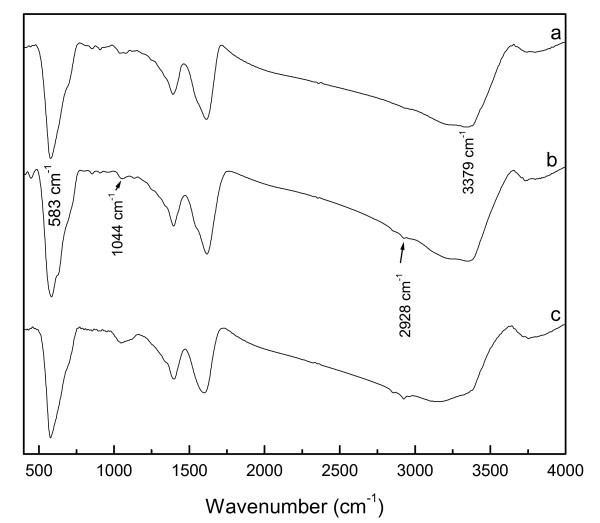
**FT-IR spectra of the Fe_3_O_4_@PPy deposits prepared under different amounts of pyrrole monomer at 160°C**: (a) 0.1 mL, (b) 0.3 mL, (c) 0.5 mL.

Figure 3 shows the FT-IR spectra of the as-prepared Fe_3_O_4_@PPy samples with different amounts of pyrrole monomer. The absorption band at 583 cm^-1 ^is attributed to Fe-O bond, the N-H stretching band of a pyrrole appears at about 3379 cm^-1^, a weak band at 2928 cm^-1 ^in Figure 3b,c is due to the C-H stretching vibration absorption. Other spectra at 1650-1029 cm^-1 ^also show the characteristic polypyrrole absorption [17]. A weak absorption at about 1620 cm^-1 ^is assigned to the C = C ring stretching of pyrrole. The C-N ring stretching band of pyrrole occurs at 1044 cm^-1^. These results demonstrated that polypyrrole was synthesized.

**Figure 4:**
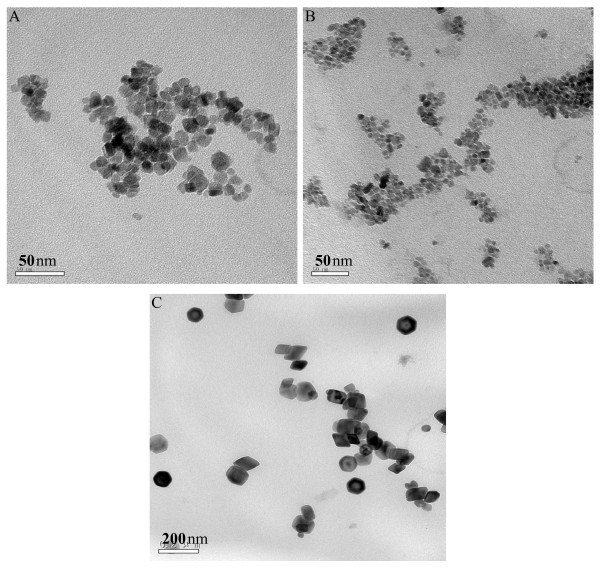
**TEM images of the Fe_3_O_4_@PPy precipitates collected by magnetic separation from the nanofluids prepared under different amounts of pyrrole monomer at 160°C**: **(A) **0.1 mL, **(B) **0.3 mL, **(C) **0.5 mL.

**Figure 5:**
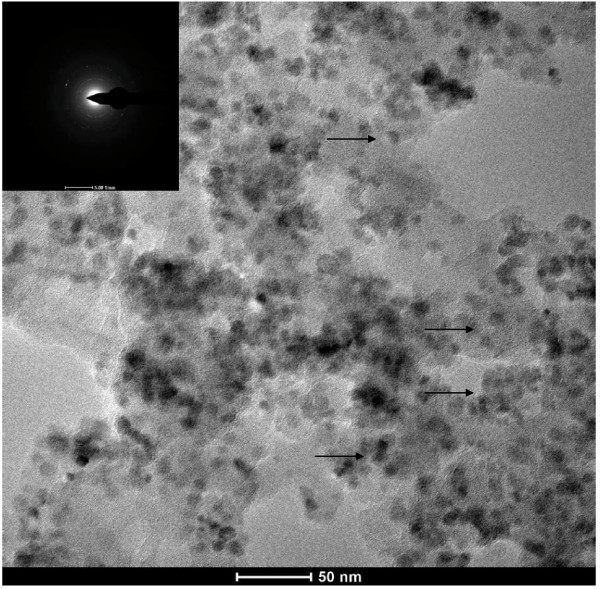
**TEM image and the corresponding SAED pattern of the Fe_3_O_4_@PPy deposit collected by magnetic separation from the nanofluid prepared at 160°C with 0.3 mL pyrrole monomer**.

The size and shape of the precipitates were determined by TEM. Figure 4 presents the typical TEM images of the precipitates produced under various amounts of pyrrole monomer. All of these precipitates present uniform sizes and morphologies. Figure 4a shows the Fe_3_O_4_@PPy NPs prepared with 0.1 mL pyrrole monomer with an average diameter of 15 nm. When the amount of pyrrole monomer is 0.3 mL, the mean diameter of the NPs is about 7 nm as shown in Figure 4b. When the amount of pyrrole is increased to 0.5 mL, the average diameter of the NPs is about 70 nm as shown in Figure 4c. Core-shell structures can be found in Figure 4a, especially in Figure 4c. This kind structure was not clearly found in Figure 4b because of small particles produced in the experimental conditions. In order to study the structure of the sample shown in Figure 4b, a magnified figure was applied. Figure 5 shows the magnified image of the sample synthesized with 0.3 mL pyrrole, in which core-shell structure was clearly found (as arrows' directions). The corresponding SAED pattern as the inset in Figure 5 shows amorphous phase. Based on the result obtained from Figure 2b, Fe_3_O_4 _was produced. Thus, we thought that Fe_3_O_4 _NPs were coated by amorphous PPy. These results demonstrated that Fe_3_O_4_@PPy NPs were obtained in the present conditions. At the same time, the formation of PPy affected the growth of Fe_3_O_4 _NPs as shown in Figure 4. When the amount of pyrrole monomer is small, such as 0.1 mL pyrrole added into the reaction system, the formation rate of the PPy is slower and the shell of PPy is thinner than those produced with higher amounts of the pyrrole, such as 0.3 and 0.5 mL pyrrole. The Fe_3_O_4 _NPs grow quickly with small amounts of pyrrole. When the amount of pyrrole monomer is too high, such as 0.5 mL pyrrole added into the reaction system, the formation rate of the PPy is faster and the shell of PPy is thicker than those produced with smaller amounts of the pyrrole. The particle size becomes bigger because of the formation of the thick PPy shell. This kind of shell can be clearly found in Figure 4c. When the amount of the pyrrole was 0.3 mL, the size of the as-produced particle is the smallest in the present experimental conditions.

### Magnetic property of the as-prepared samples

**Figure 6:**
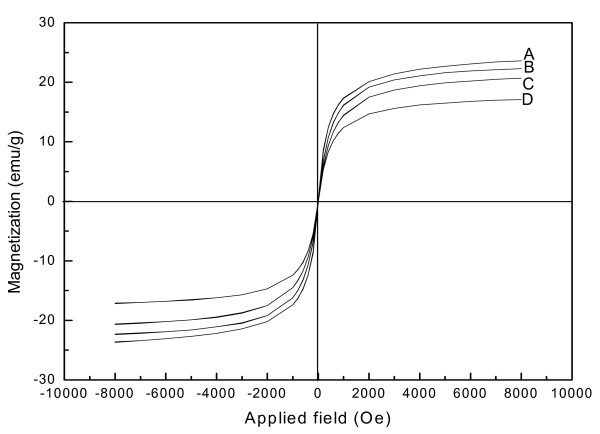
**Magnetization curve of the Fe_3_O_4_@PPy prepared under different amounts of pyrrole monomer at 160°C**: (A) 0 mL, (B) 0.1 mL, (C) 0.3 mL, (D) 0.5 mL.

Magnetic properties of the as-prepared products were investigated by a VSM at room temperature in the applied magnetic field from -8 to 8 KOe. The hysteresis loops of the Fe_3_O_4_@PPy NPs are shown in Figure 6. The products showed typical superparamagnetic behavior with negligible coercivity and remanence. Superparamagnetic behavior is often observed at room temperature with Fe_3_O_4 _NPs smaller than 20 nm [18,19]. In order to study the effect of the PPy shell on magnetic property of the samples, the hysteresis loop of the Fe_3_O_4 _NPs is shown as Figure 6A, in which the Fe_3_O_4 _NPs were prepared without any pyrrole and other experimental conditions kept the same. The saturation magnetizations of the as-prepared products were determined to be 23.7, 22.4, 20.7, and 17.2 emu/g for the samples fabricated with 0, 0.1, 0.3, and 0.5 mL pyrrole, respectively. These data shown that the saturation magnetization became smaller with increase of pyrrole added into the reaction system, for which the shell of the as-prepared shell-core samples became thicker with increase of pyrrole. These results demonstrated that Fe_3_O_4_@PPy NPs were produced.

### Effects of trisodium citrate (Na_3_cit) on the structure of the sample

**Figure 7:**
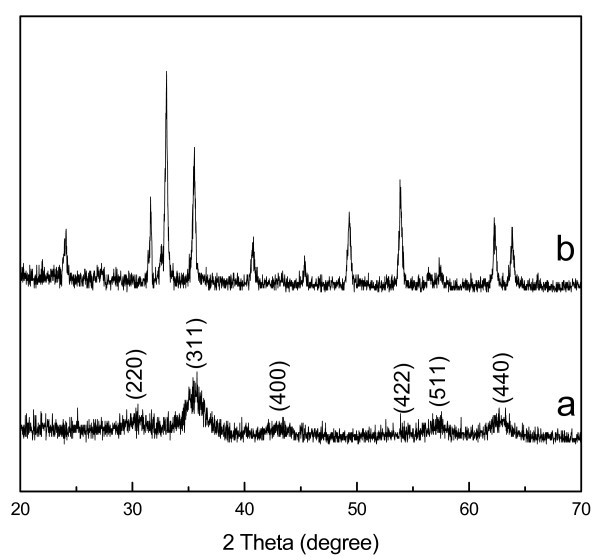
**XRD diffraction patterns of the solid samples prepared under solutions**: (a) 9 mL 0.06 M Na_3_cit aqueous solution, (b) 9 mL water.

In order to study the effects of Na_3_cit on the structure of the product, different amounts of Na_3_cit were used and other experimental conditions kept the same in the reaction systems. Figure 7 shows XRD patterns of the as-prepared samples under 9 mL 0.06 M Na_3_cit aqueous solution and 9 mL water instead of the 9 mL 0.1 M Na_3_cit aqueous solution as described in the "Experimental" section, respectively. When the amount of the Na_3_cit was decreased from 9 mL 0.1 M to 9 mL 0.06 M Na_3_cit aqueous solution, pure Fe_3_O_4 _was also obtained as shown in Figure 7a. When no Na_3_cit was added into the solution, almost pure α-Fe_2_O_3 _was produced as shown in Figure 7b. These results demonstrated that the Na_3_cit was used as the reducing reagent to form Fe_3_O_4_.

**Figure 8:**
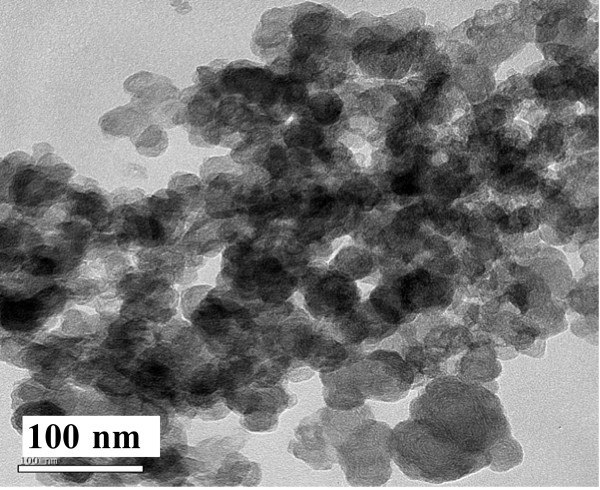
**TEM image of the solid sample prepared with 9 mL 0.06 M Na_3_cit aqueous solution**.

The size and shape of the product prepared in 9 mL 0.06 M Na_3_cit aqueous solution were determined by TEM as shown in Figure 8, in which the mean diameter of the particles is about 30 nm. Compared with the product prepared in 9 mL 0.1 M Na_3_cit aqueous solution as shown in Figure 4b, the size of the sample increased from 7 to 30 nm with the decrease of the amount of Na_3_cit in the solution. These results were in agreement with references [20,21], in which Na_3_cit was used as a dispersant.

### Formation mechanism of the stable fluid

**Figure 9:**
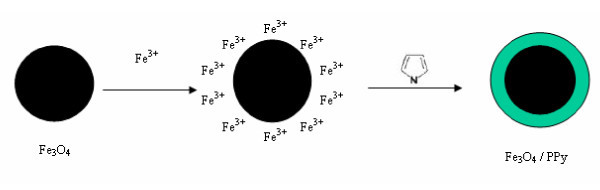
**Scheme of the formation processes of the as-produced Fe_3_O_4_@PPy NPs**.

In the present conditions, the Na_3_cit acted as a reductant and a dispersant. The formation processes of the Fe_3_O_4 _NPs are listed in Equations 1-5. When the Fe_3_O_4 _NPs were produced, the Fe^3+ ^ions were absorbed on the surface of the Fe_3_O_4 _NPs. The polymerizing reaction of the pyrrole monomers took place on the surface of the Fe_3_O_4 _NPs, in which the Fe^3+ ^ions were used as an initiator. So the PPy coated the magnetite nanoparticles. The forming processes are shown in Figure 9. At the same time, these processes prevent the Fe_3_O_4 _growing. Thus, this polymerizing reaction can be applied to control the size of the as-prepared sample.(1)(2)(3)(4)(5)

A steric stabilization effect is always used to explain the stability of particles suspended in the base fluid [22]. This steric stabilization effect arises from the fact that polymers coating on the surface of NPs occupy a certain amount of space. Thus, the space becomes compressed when nanoparticles are brought too close together. An associated repulsive force makes separate nanoparticles from each other and restrains the aggregation of nanoparticles. On the other hand, the weight of the NPs affected the stability of the nanofluids as shown in Figure 1.

In the present experiments, SDS was also used as a dispersant. The size of the Fe_3_O_4 _NPs became smaller with SDS (these results are not shown here). Compared with surfactants, the steric effect by polymers onto the surface of NPs is a better way to stabilize NPs suspended in the based fluid [2]. The PPy-coated Fe_3_O_4 _NPs have the steric stabilization effect and achieve a better and larger solubility of NPs in water because of the solubility rule of similarity. Thus, stable nanofluids containing magnetic Fe_3_O_4 _NPs can be produced with the help of PPy.

The nanofluids containing Fe_3_O_4_@PPy NPs can be used to enhance heat transfer, as magnetic nanofluids and other special applications, as PPy is one of the conducting polymers. This facile method can be used to prepare other organic-inorganic NPs.

## Conclusion

A facile and novel method for preparing nanofluids containing Fe_3_O_4_@PPy was presented, in which one-pot route was used. The nanofluid can keep long-term stability and very good dispersing. Fe_3_O_4_@PPy NPs can still keep dispersing well after the nanofluid has been standing for 1 month and no sedimentation is found. The Na_3_cit acted as a reductant and a dispersant to form Fe_3_O_4 _NPs. Fe^3+ ^ions remained in the solution adsorbed on the surface of the Fe_3_O_4 _NPs. The polymerization reaction of the pyrrole monomers took place with these Fe^3+ ^ions as the initiator. The particle size of the as-prepared Fe_3_O_4_@PPy can be easily controlled from 7 to 30 nm by adjusting the amount of pyrrole monomer. The steric stabilization and weight of the NPs affect the stability of the nanofluids. The as-prepared Fe_3_O_4_@PPy NPs exhibit superparamagnetic behavior with various saturation magnetizations.

## Abbreviations

PPy: Polypyrrole; NPs: nanoparticles; XRD: X-ray powder diffraction; TEM: transmission electron microscopy; VSM: vibrating sample magnetometer.

## Competing interests

The authors declare that they have no competing interests.

## Authors' contributions

**BZ **performed experiments and helped to draft the manuscript. ZN proposed idea, designed experiments and finalized the manuscript. All authors read and approved the manuscript.

## Note

**Figure 1. XRD patterns of the as-synthesized Fe_3_O_4 _NPs**.

**Figure 2. (a) **Survey XPS spectrum of the as-synthesized Fe_3_O_4 _NPs. **(b) **High-resolution XPS Fe_2p _spectrum.

**Figure 3. TEM images of the Fe_3_O_4 _NPs**.

## Supplementary Material

Additional file 1 **In a typical experiment, NaOH 0.0800 g (2.0 mmol) and 0.0230 g sodium dodecyl sulfate (0.008 M) were dissolved into a 9 mL aqueous solution containing 0.1 M Trisodium citrate (C_6_H_5_Na_3_O_7 _2H_2_O) under constant stirring**. 1.0 mL of FeCl_3 _solution (1.0 M) was added into the solution. The whole mixture was stirred vigorously for 5 min to give a homogeneous solution. Subsequently, the solution was transferred into a 50 mL Teflon-lined stainless steel autoclave, and maintained at 160°C for 24 h. Afterward, the autoclave was allowed to cool down to room temperature naturally. The deposit was obtained by magnetic separation method, and washed with distilled water and absolute ethanol for several times. The as-prepared black precipitate was dried under vacuum at 50°C for 24 h. The crystalline structure and chemical composition of the product were determined by XRD analyses as shown in Figure 1. The positions and relative intensities of all diffraction peaks can be well indexed to the magnetic cubic structure of Fe_3_O_4_. The XPS spectrum of the product is shown in Figure 2a. The detail spectrum of the sample corresponding to the binding energies of Fe2p is shown in Figure 2b. The photoelectron peaks at 710.9 and 724.4 eV correspond to Fe 2p3/2 and Fe 2p1/2, respectively. These results are in concert to previous report [1]. The size and shape of the products were determined by TEM. Figure 3 shows the typical TEM images of the Fe_3_O_4 _prepared with various concentrations of Na_3_cit. Click here for file
