# Supplementary material for: Preparation of stable magnetic nanofluids containing Fe3O4@PPy nanoparticles by a novel one-pot route
Source: Nanoscale Res Lett. 2011 Mar 16;6(1):230. doi: 10.1186/1556-276X-6-230 (PMC3211289; doi:10.1186/1556-276X-6-230)
Supplement: Additional file 1 — In a typical experiment, NaOH 0.0800 g (2.0 mmol) and 0.0230 g sodium dodecyl sulfate (0.008 M) were dissolved into a 9 mL aqueous solution containing 0.1 M Trisodium citrate (C6H5Na3O7 2H2O) under constant stirring. 1.0 mL of FeCl3 solution (1.0 M) was added into the solution. The whole mixture was stirred vigorously for 5 min to give a homogeneous solution. Subsequently, the solution was transferred into a 50 mL Teflon-lined stainless steel autoclave, and maintained at 160°C for 24 h. Afterward, the autoclave was allowed to cool down to room temperature naturally. The deposit was obtained by magnetic separation method, and washed with distilled water and absolute ethanol for several times. The as-prepared black precipitate was dried under vacuum at 50°C for 24 h. The crystalline structure and chemical composition of the product were determined by XRD analyses as shown in Figure 1. The positions and relative intensities of all diffraction peaks can be well indexed to the magnetic cubic structure of Fe3O4. The XPS spectrum of the product is shown in Figure 2a. The detail spectrum of the sample corresponding to the binding energies of Fe2p is shown in Figure 2b. The photoelectron peaks at 710.9 and 724.4 eV correspond to Fe 2p3/2 and Fe 2p1/2, respectively. These results are in concert to previous report [1]. The size and shape of the products were determined by TEM. Figure 3 shows the typical TEM images of the Fe3O4 prepared with various concentrations of Na3cit. [file 1556-276X-6-230-S1.DOC]

**Supporting Information**

In a typical experiment, NaOH 0.0800 g (2.0 mmol) and 0.0230 g sodium dodecyl sulfate (0.008 M) was dissolved into a 9 mL aqueous solution containing 0.1 M Trisodium citrate (C_6_H_5_Na_3_O_7_·2H_2_O) under constant stirring. 1.0 mL of FeCl_3_ solution (1.0 M) was added into the solution. The whole mixture was stirred vigorously for 5 min to give a homogeneous solution. Subsequently, the solution was transferred into a 50 mL Teflon-lined stainless steel autoclave, and maintained at 160 ^○^C for 24 h. Afterward, the autoclave was allowed to cool down to room temperature naturally. The deposit was obtained by magnetic separation method, and washed with distilled water and absolute ethanol for several times. The as-prepared black precipitate was dried under vacuum at 50 ^○^C for 24 h.

The crystalline structure and chemical composition of the product was determined by XRD analyses as shown in Figure 1. The positions and relative intensities of all diffraction peaks can be well indexed to the magnetic cubic structure of Fe_3_O_4_. The XPS spectrum of the product is presented in Figure 2a. The detail spectrum of the sample corresponding to the binding energies of Fe2p is shown in Figure 2b. The photoelectron peaks at 710.9 and 724.4 eV were corresponds to Fe 2p3/2 and Fe 2p1/2, respectively. These results are in concert to previous report [1]. The size and shape of the products were determined by TEM. Figure 3 presents the typical TEM images of the Fe_3_O_4_ prepared with various concentrations of Na_3_Cit.

**Reference**

1 G. C. Xi, C. Wang, X. Wang, Eur. J. Inorg. Chem. 425 (2008).


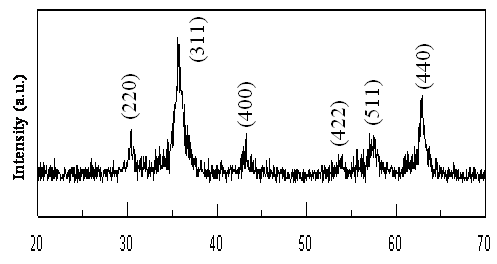


**Figure 1.** XRD patterns of the as-synthesized Fe_3_O_4_ NPs.

$$a$$

$$b$$

**Figure 2.** (a) Survey XPS spectrum of the as-synthesized Fe_3_O_4_ NPs, (b) High-resolution XPS Fe_2p_ spectrum.


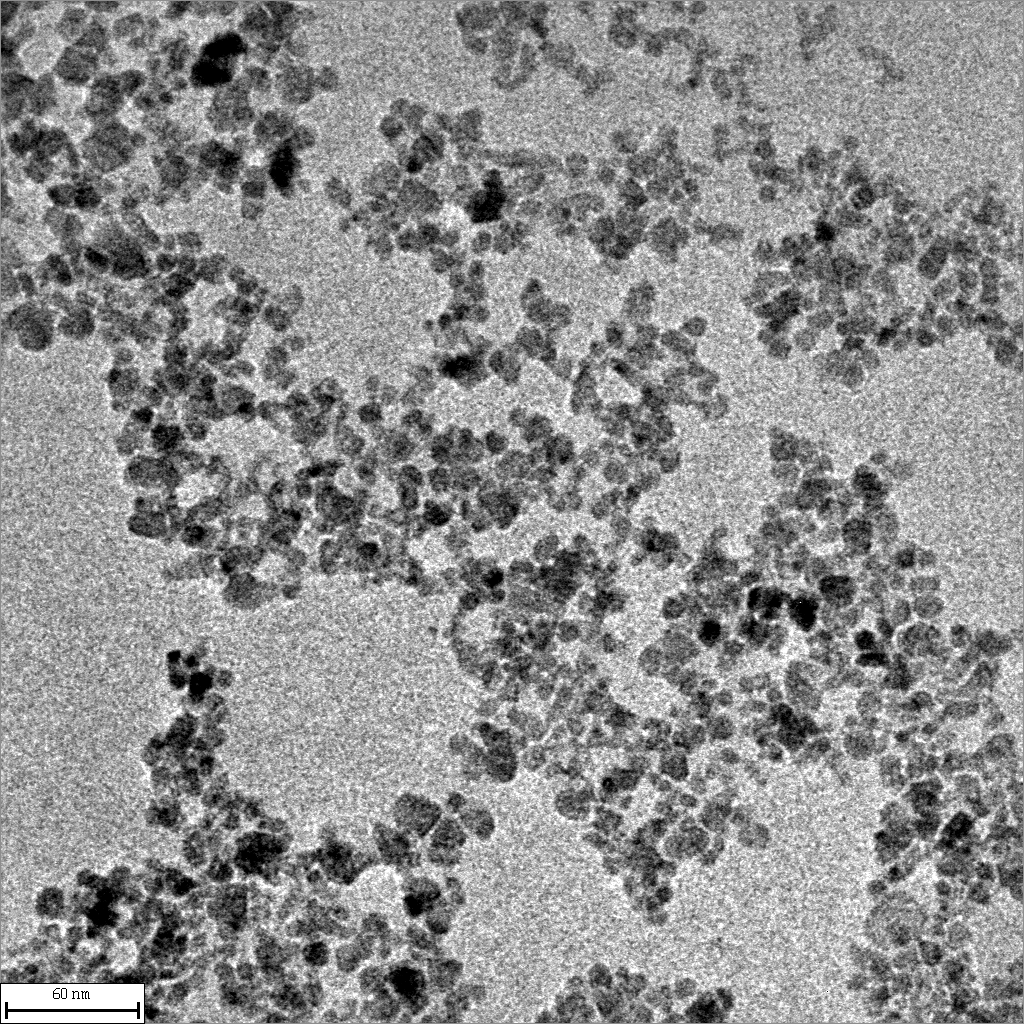


**Figure 3.** TEM images of the Fe_3_O_4_ NPs.
